# Volumetry improves the assessment of the vestibular aqueduct size in inner ear malformation

**DOI:** 10.1007/s00405-022-07681-4

**Published:** 2022-10-10

**Authors:** Nora M. Weiss, Tabita M. Breitsprecher, Alexander Pscheidl, David Bächinger, Stefan Volkenstein, Stefan Dazert, Robert Mlynski, Sönke Langner, Peter Roland, Anandhan Dhanasingh

**Affiliations:** 1 Department of Otorhinolaryngology-Head and Neck Surgery, Ruhr-University Bochum, St. Elisabeth-Hospital Bochum, Bochum, Germany; 2 Department of Translational Neurosciences, Faculty of Medicine and Health Sciences, University of Antwerp, Antwerp, Belgium; 3 Department of Otorhinolaryngology, Head and Neck Surgery, Medical Center, Dortmund, Germany; 4 Department of Otorhinolaryngology, Head and Neck Surgery, University Hospital Zurich, Zurich, Switzerland; 5 University of Zurich, Zurich, Switzerland; 6 Department of Otorhinolaryngology, Head and Neck Surgery, “Otto Körner”, Rostock University Medical Center, Rostock, Germany; 7 Institute of Diagnostic and Interventional Radiology, Pediatric and Neuroradiology, Rostock University Medical Center, Rostock, Germany; 8 Department of Otolaryngology-Head and Neck Surgery and Neurological Surgery, University of Texas, Southwestern Medical Center, Dallas, TX USA; 9 MED-EL, Innsbruck, Austria

**Keywords:** Cochlear malformation, Inner ear malformation, Diagnosis, Volume, 3D segmentation

## Abstract

**Objectives:**

Enlarged vestibular aqueduct (EVA) is a common finding associated with inner ear malformations (IEM). However, uniform radiologic definitions for EVA are missing and various 2D-measurement methods to define EVA have been reported. This study evaluates VA volume in different types of IEM and compares 3D-reconstructed VA volume to 2D-measurements.

**Methods:**

A total of 98 high-resolution CT (HRCT) data sets from temporal bones were analyzed (56 with IEM; [cochlear hypoplasia (CH; *n* = 18), incomplete partition type I (IPI;* n* = 12) and type II (IPII;* n* = 11) and EVA (*n* = 15)]; 42 controls). VA diameter was measured in axial images. VA volume was analyzed by software-based, semi-automatic segmentation and 3D-reconstruction. Differences in VA volume between the groups and associations between VA volume and VA diameter were assessed. Inter-rater-reliability (IRR) was assessed using the intra-class-correlation-coefficient (ICC).

**Results:**

Larger VA volumes were found in IEM compared to controls. Significant differences in VA volume between patients with EVA and controls (*p* < 0.001) as well as between IPII and controls (*p* < 0.001) were found. VA diameter at the midpoint (VA midpoint) and at the operculum (VA operculum) correlated to VA volume in IPI (VA midpoint: *r* = 0.78, VA operculum: *r* = 0.91), in CH (VA midpoint: *r* = 0.59, VA operculum: *r* = 0.61), in EVA (VA midpoint: *r* = 0.55, VA operculum: *r* = 0.66) and in controls (VA midpoint: *r* = 0.36, VA operculum: *r* = 0.42). The highest IRR was found for VA volume (ICC = 0.90).

**Conclusions:**

The VA diameter may be an insufficient estimate of VA volume, since (1) measurement of VA diameter does not reliably correlate with VA volume and (2) VA diameter shows a lower IRR than VA volume. 3D-reconstruction and VA volumetry may add information in diagnosing EVA in cases with or without additional IEM.

## Introduction

Inner ear malformations (IEM) are responsible for approximately 20–30% of cases with congenital profound sensorineural hearing loss (SNHL) [1–4]. The therapy of choice for patients with profound SNHL associated with IEM usually consists of cochlear implantation (CI). In IEM, in addition to the anatomy of the cochlea and the auditory nerve, particular attention should be paid to the radiologic morphology of the vestibular aqueduct (VA) [5]. Enlarged vestibular aqueduct syndrome (EVAS) is the most common IEM in children with SNHL [6]. The bony VA harbors the intraosseous portion of the endolymphatic sac and the endolymphatic duct, which connects the endolymphatic sac to the endolymphatic system of the cochlea and the vestibular labyrinth. Although the functional significance of the endolymphatic duct and sac is poorly understood, it is hypothesized that these structures critically contribute the inner ear fluid and electrolyte homeostasis. An enlarged VA (EVA) develops due to an enlarged, dysfunctional endolymphatic sac and duct as a consequence of a complex inner ear endothelial dysfunction perturbating the endolymph composition [7, 8]. EVA may be a risk factor for vestibular symptoms and hearing loss, and may be associated with syndromic disorders [9]. The pathophysiology of audiovestibular symptoms associated with EVAS is not well understood. The endolymphatic sac dysfunction or additional ion transport pathologies of the inner ear may explain both the episodic cochleovestibular symptoms as well as the progressive sensorineural hearing loss due to neurosensory degeneration [7, 10]. Moreover, a conductive hearing loss commonly observed in EVAS is likely to be the cause of a third window effect of the EVA [11]. Furthermore, an EVA is likely to be associated with modiolar defects resulting in cerebrospinal fluid (CSF) leaks (“CSF gusher”; [12–14]). CSF gusher is accompanied by peri-/intraoperative challenges to seal the cochlea and can lead to postoperative meningitis [15]. Moreover, recent studies demonstrated an association between the risk of postoperative vertigo in patients with EVA and with simultaneously increased endolymphatic sac volume [16]. Preoperative assessment of VA size could therefore be useful in improving perioperative management and assist in perioperative risk reduction. For this reason, preoperative computed tomography (CT) and/or magnetic resonance imaging (MRI) are performed routinely to determine morphological abnormalities which could affect treatment, particularly surgical intervention, and to prognosticate hearing outcome. Nevertheless, imaging may be challenging to interpret [17].

EVA is commonly diagnosed on axial CT images by measuring the diameter of the VA in the middle of its course or at the level of the operculum. However, uniform definitions for EVA are missing and various 2D measurement methods to define EVA are reported in the literature. The Valvassori criteria define an enlarged VA as larger than 1.5 mm measured at the midpoint of the VA [18]. A more recent classification (“Cincinnati criteria”) includes two different measurement points, one at the midpoint and one at the operculum [19]. In a further classification, Weissman suggests using the diameter of the adjacent semicircular canal as a reference [20]. Dewan et al. compared two different classification systems and found varying results (44% versus 16% EVAS) depending on the criteria used [21]. In addition, a high variability in 2D VA diameter measurements among the axial, oblique and double oblique view in CT was reported [22]. In this study, it is hypothesized that 3D reconstruction of the VA facilitates the evaluation of VA size and that volumetric data may be useful as a diagnostic criterion for EVA. The aim of this study was to determine VA diameter and volume in different types of IEM. Furthermore, we studied whether 2D parameters, such as VA diameter, correlate with VA volume and whether VA volumetry may support the use of a specific 2D measurement cutoff value for the diagnosis of EVA.

## Methods

### Image analysis

**Fig. 1:**
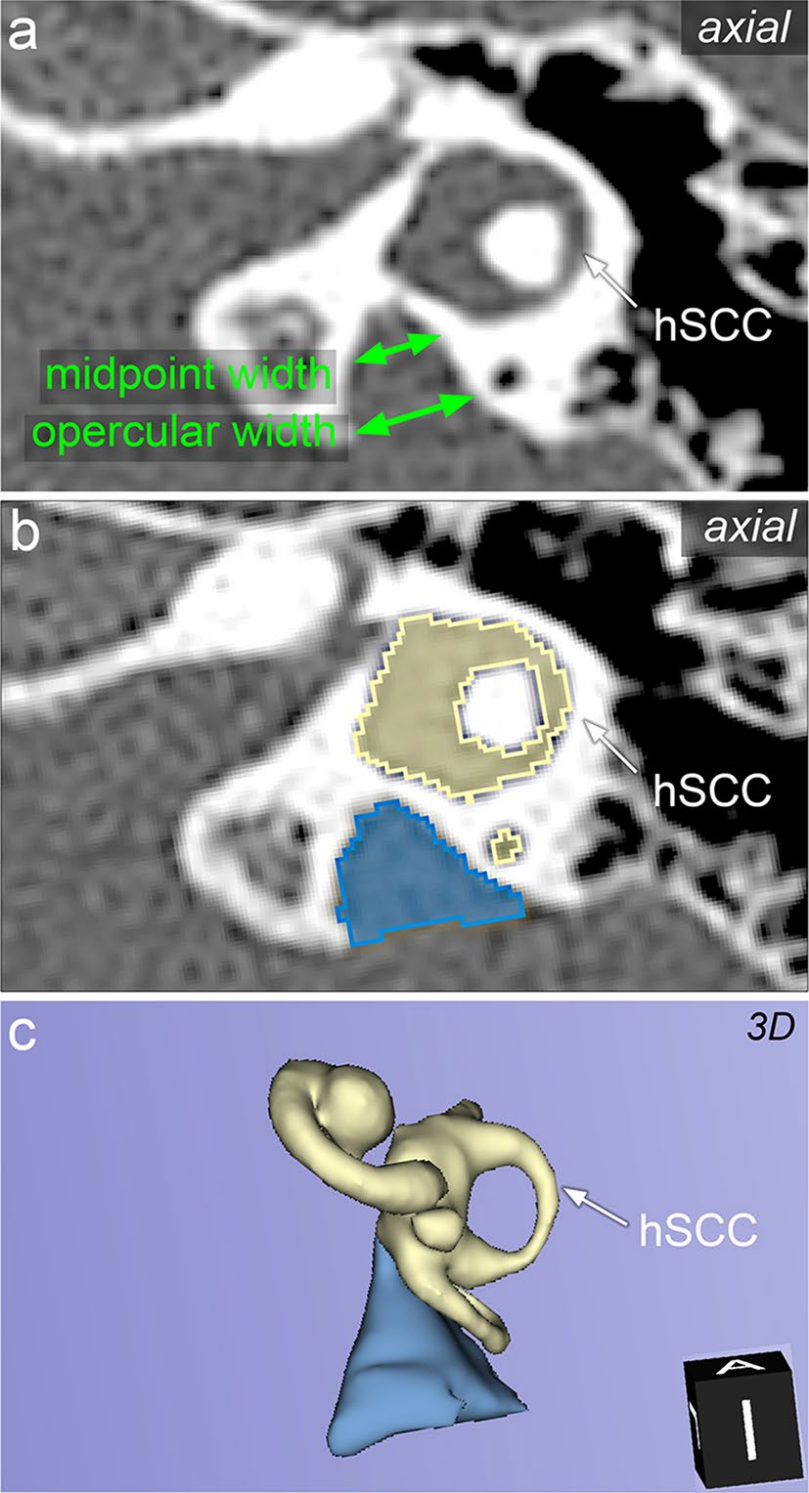
Exemplary measurements of the vestibular aqueduct (VA) width and 3D reconstruction in a temporal bone with an enlarged VA (EVA). **a** Two-dimensional measurements according to the Cincinatti criteria at the midpoint (upper green arrow) and the operculum (lower green arrow) of the VA in an axial high-resolution computed tomography (HRCT). **b** Segmentation of the 3D reconstruction from the axial HRCT. Yellow: segmentation of the bony labyrinth. Blue surface: segmentation of the VA. **c** Three-dimensional reconstruction of the bony labyrinth (yellow) and the EVA (blue). *hSCC*, horizontal semicircular canal

In CT data sets, multiplanar slices were reconstructed in the axial plane (0.1–1 mm). The diameter of the VA was determined at the midpoint between the VA exit from the vestibule and the operculum (VA_midpoint_) as well as at the level of the operculum (VA_operculum_) as suggested by Boston et al. [19] (Fig. 1a). The definition of EVA was based on the Cincinnati criteria with operculum width > 1.9 mm and/or midpoint width > 0.9 mm [19] and was measured according to Wang et al. [23].

Furthermore, the CT data sets were reconstructed using 3D slicer (https://www.slicer.org/, version 4.13.0, Massachusetts, USA [24]). Segmentation of the inner ear was performed using threshold analysis (threshold range − 1024 to 700 Hounsfield units) and a 3D model of the inner ear was reconstructed as described elsewhere [25] (Fig. 1b). The VA volume was calculated using the segmentation module and the segment statistics module in the 3D slicer software (Fig. 1c). IEM were diagnosed according to the Sennaroglu and Saatci classification [2, 26]. In inconclusive cases, the INCAV criteria were added [27]. All measurements were performed by two independent examiners (ENT residents with more than 1 year of experience interpreting temporal bone imaging after instruction and training under the supervision of two senior physicians [radiology consultant and ENT consultant, each with more than 6 years of experience]). Both investigators were blinded to the previous measurement. All CT data sets were anonymized prior image analyses. The study was approved by the local ethics committee (No. A2019-0201).

### Statistical analysis

Statistical analyses were performed using Prism (version 8, GraphPad Software, La Jolla, CA, USA). The significance level was set to *p* < 0.05. Normal distribution was tested using the Kolmogorov–Smirnov test. Data did not pass normality test. To compare differences among groups, the Kruskal–Wallis test was used. Dunn’s test was used to correct for multiple comparisons. Correlations were assessed using Spearman correlation. The inter-rater reliability (IRR) was determined by calculating the intra-class correlation coefficient (ICC). Receiver operating characteristic (ROC) curves were determined to estimated sensitivity and specificity. The cutoff value was determined, where Youden's index, i.e. sensitivity + specificity − 1, reached its maximum.

## Results

In this retrospective multi-center study, 56 high-resolution CT (HRCT) of the temporal bone from patients undergoing cochlear implantation due to severe to profound sensorineural hearing loss because of IEM were analyzed. All CT data sets were reconstructed using a hard kernel and bone window/level setting. Slice thickness varied between 0.625 and 1 mm. IEM consisted of 15 cases of EVAS, 18 cases of cochlear hypoplasia (CH), 12 cases of incomplete partition (IP) type I (IPI) and 11 cases of IP type II (IPII) with EVA (Mondini malformation). HRCT data sets of 42 patients with no inner ear pathology, including no SNHL and no prior ear surgery were used as a reference.

Based on the Cincinnati criteria, 2D measurements showed EVA defined as an VA_operculum_ > 1.9 mm in 35/56 (63%) IEM cases. In all these 35 cases, the VA_midpoint_ was > 0.9 mm. In addition, 9/56 (16%) cases showed EVA defined by a VA_midpoint_ > 0.9 mm. Taken together, a total of 44/56 (79%) cases exhibited EVA according to the Cincinnati criteria. The range of values for the diameter of the VA_midpoint_ and the VA_operculum_ for the individual malformation types is shown in Table 1. The control group showed an VA_operculum_ < 1.9 mm and a VA_midpoint_ ≤ 0.9 in all cases.

**Table 1:** Descriptive statistics of three-dimensional and two-dimensional measurements. Volume and diameter of the VA_midpoint_ and the VA_operculum_ for the individual malformation types

	Operculum_control_	Midpoint_control_	Operculum_CH_	Midpoint_CH_	Operculum_IPI_	MidpointI_PI_	Operculum_IPII_	Midpoint_IPII_	Operculum_EVAS_	Midpoint_EVAS_
Number of cases	43	43	18	18	12	12	11	11	15	15
Minimum (mm)	0.2	0.2	0.0	0.0	0.0	0.0	2.2	1.2	1.9	1.0
25% Percentile (mm)	0.7	0.5	0.6	0.2	0.6	0.9	3.0	1.7	2.1	1.4
Median (mm)	0.9	0.6	1.1	0.8	1.4	1.6	3.4	2.1	3.4	2.2
75% Percentile (mm)	1.0	0.7	3.6	2.3	2.7	2.0	4.1	2.3	4.1	2.9
Maximum (mm)	1.4	1.1	7.0	3.4	3.3	2.2	4.8	3.1	5.4	3.8

**Fig. 2:**
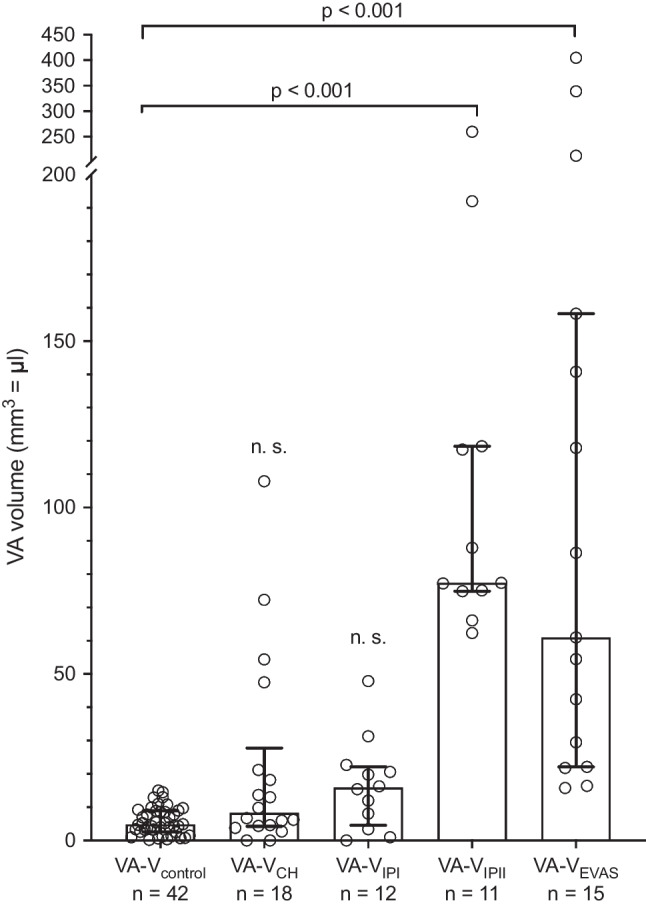
Scatterplot showing the distribution of values of vestibular aqueduct volume (VA-V) of the individual inner ear malformation (IEM) groups and the VA-V_control_. Significant differences between the control group and the VA-V_EVA_ as well as between the control group and the VA-V_IPII_ were found. *n.s.* not significant. Box indicates median, whiskers indicate interquartile range. The upper section of the *y*-axis has been compressed for better visualization

A 3D model of the inner ear was successfully reconstructed in every case. The procedure of segmentation, reconstruction and volume determination takes approximately 25 min. The values for individual VA volumes (VA-V_control_ = VA volume control; VA-V_CH_ = VA volume CH; VA-V_IPI_ = VA volume IPI; VA-V_IPII_ = VA volume IPII; VA-V_EVAS_ = VA volume EVAS) among the different IEM are shown in Fig. 2. The median VA volume was higher in all IEM types compared to the control group. The Kruskal–Wallis test revealed significant differences in the VA volume between different groups of IEMs (5 groups, *n* = 101, *p* < 0.001). Post hoc analysis showed significant differences between VA-V_EVAS_ and VA-V_control_ (median difference 56.2 mm^3^, 95% CI 28.5–127.9 mm^3^, *p* < 0.001) as well as between VA-V_IPII_ and VA-V_control_ (median difference 72.6 mm^3^, 95% CI 68.6–109.8 mm^3^, *p* < 0.001). VA-V_CH_ and VA-V_IPI_ exhibited a trend to a larger values than VA-V_control_ (VA-V_CH_: *p* = 0.24; VA-V_IPI_: *p* = 0.15) that did not reach statistical significance (Fig. 2). A vestibular aqueduct volume of > 15.4 mm^3^ differentiated EVAS from a normal vestibular aqueduct with a specificity of 100.0% (95% CI 83.2–100.0%) and a sensitivity of 100.0% (95% CI 92.1–100.0%).

**Fig. 3:**
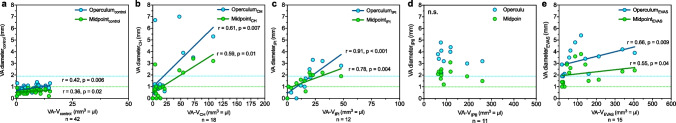
Correlations between vestibular aqueduct (VA) volume and VA diameter measured at the VA midpoint (VA_midpoint_) and at the operculum (VA_operculum_) in controls (**a)**, the cochlear hypoplasia (CH) group (**b**) the incomplete partition type I (IPI) group (**c**), the incomplete partition type II (IPII) group (**d**) and the enlarged vestibular aqueduct syndrome (EVAS) group (**e**). *r* Spearman’s rank correlation coefficient. Line represents linear regression line. The scaling of the *x*-axes differs among the groups for better visualization

Correlations between the VA volume and the diameter of the VA were found for the control group (VA_midpoint_: *r* = 0.36, *p* = 0.02, VA_operculum_: *r* = 0.42, *p* = 0.006; Fig. 3a), CH (VA_midpoint_: *r* = 0.59, *p* = 0.01, VA_operculum_: *r* = 0.61, *p* = 0.007; Fig. 3b), IPI (VA_midpoint_: *r* = 0.78, *p* = 0.004, VA_operculum_: 0.91, *p* = 0.001; Fig. 3c) and EVAS (VA_midpoint_: *r* = 0.55, *p* = 0.04, VA_operculum_: *r* = 0.66, *p* = 0.009; Fig. 3e). No correlations between the VA volume and the diameter of the VA were found for IPII (Fig. 3d). Overall, the VA_operculum_ showed a slightly stronger correlation to the VA volume compared to the VA_midpoint_.

A good inter-rater reliability was found for the 2D measurements at the operculum (ICC = 0.82) as well as at the midpoint (ICC = 0.86). The inter-rater reliability for the 3D measurements was excellent (ICC = 0.90).

**Fig. 4:**
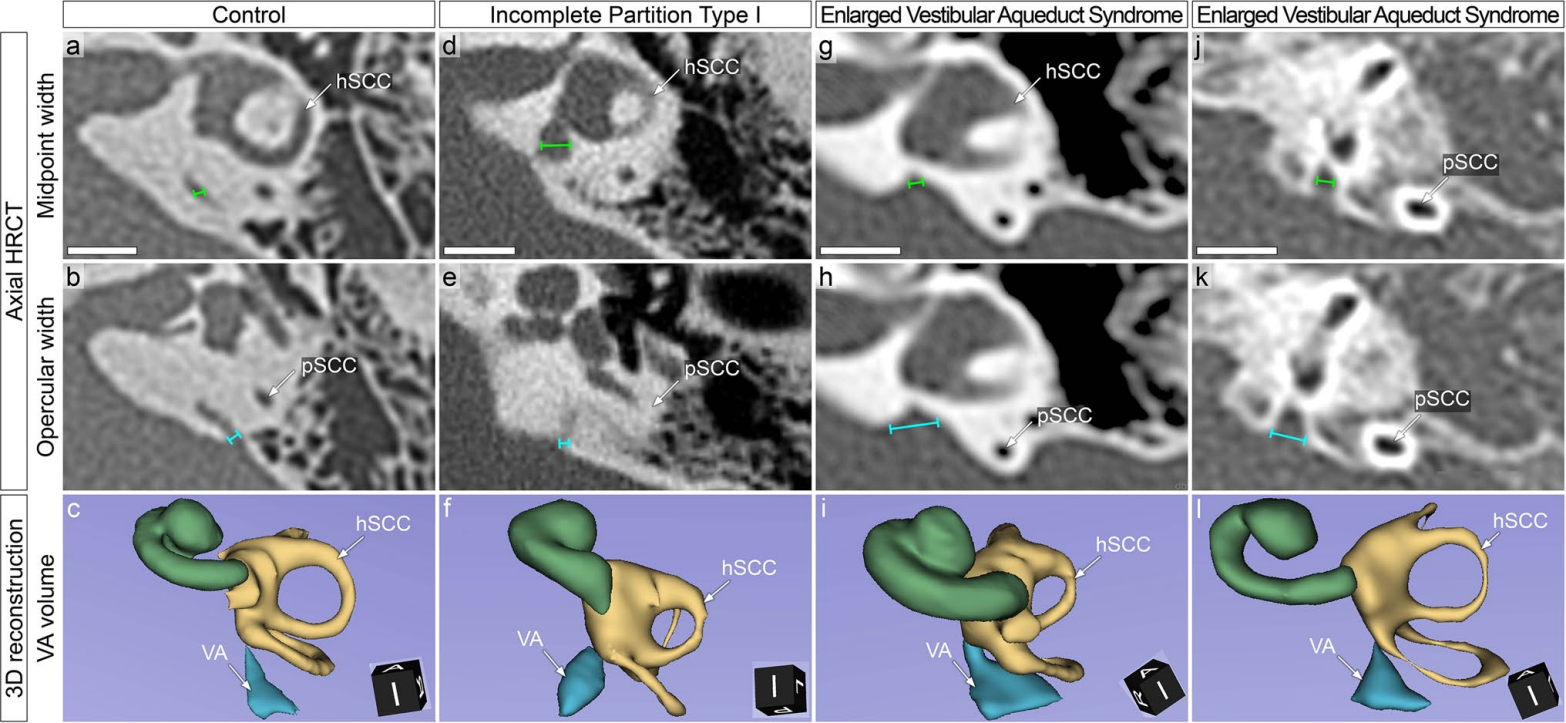
Exemplary vestibular aqueduct (VA) shapes in a control and three cases of inner ear malformation (IEM). **a–c** 2D-measurements of the VA_midpoint_ (**a**) and the VA_operculum_ (**b**) in the axial high-resolution computed tomography (HRCT) as well as three-dimensional (3D) reconstruction of the bony labyrinth (**c**) in a normal control. **d–f** Case 1, incomplete partition type I: two-dimensional (2D) measurements of the VA_midpoint_ (**d**) and VA_operculum_ (**e**) in the axial HRCT as well as a 3D reconstruction (**f**). The VA is centrally dilated with narrowing toward the operculum and the vestibule. **g–i** Case 2, enlarged vestibular aqueduct syndrome (EVAS): 2D measurements of the VA_midpoint_ (**g**) and VA_operculum_ (**h**) in the axial HRCT as well as 3D reconstruction of the bony labyrinth (**i**). **j–l** Case 3, EVAS: 2D-measurements of the VA_midpoint_ (**j**) and VA_operculum_ (**k**) in the axial HRCT as well as a 3D reconstruction of the bony labyrinth (**l**). In both case 2 and case 3, the VA is narrow toward the vestibule and shows a very broad opening toward the operculum. Both cases were classified as EVA according to the Cincinnati criteria (case 2: VA_midpoint_ = 1.0 mm, VA_operculum_ = 2.1 mm; case 3: VA_midpoint_ = 1.0 mm; VA_operculum_ = 1.9 mm). The volume of case 2 is 29.5 mm^3^. The volume of case 3 is 16.4 mm^3^. Green volume, cochlea; yellow volume: vestibular labyrinth; blue volume: VA. *hSCC* horizontal semicircular canal, *pSCC* posterior semicircular canal. Scale bars: 5 mm

Highly varying shapes of the VA in different types of IEM were observed. Figure 4 shows exemplary VA shapes in a control (Fig. 4a–c) and a case of IEM with central dilatation and narrowing toward the operculum and the vestibule (Fig. 4d–f). Furthermore, two cases with both very wide VA openings toward the operculum, but contrasting VA volumes are shown (Fig. 4g–l).

## Discussion

The results of the present study suggest that VA diameter alone may be an insufficient estimate of the total VA volume, since first, measurement of VA diameter does not correlate well with VA volume, and second, the VA diameter shows a lower IRR than VA volume. In particular, we found that VA diameter does not accurately predict VA volume among EVA cases. Inconsistent correlations were found between the VA volume and the VA diameter. We introduced volumetry of the VA based on radiologic data as a novel diagnostic approach for EVA. Using VA volumetry, we found a high prevalence of EVA in several types of IEM. Diagnosing EVA is hindered by abnormal VA shapes, which challenge 2D measurements from axial CT images. Measuring VA diameter may lead to highly varying results depending on the imaging quality, the chosen slice and the individual investigator. This may be another explanation for by a majority moderate correlations between the VA volume and the VA diameter in measurements of the VA in HRCTs.

The VA is commonly evaluated on axial images. However, the exact position along the VA, where the VA diameter should be determined, is a matter of debate. The first to propose VA measurements, Valvassori and Clemis, considered a VA with a width of on axial view greater than 1.5 mm at the midpoint of its course from the vestibule to the posterior cranial fossa as enlarged [18]. Later, other authors introduced different criteria and used measurements at the operculum [19]. However, widely accepted definitions are missing and current measurement methods may lead to misdiagnoses, since they only consider a maximum of two measurement points [12]. Yet, the identification and accurate measurement of the VA is challenging even in subjects with normal anatomy and a correct assessment of VA volume. Consequently, this is even more difficult in patients with IEM [28–30]. The present study provides evidence that measuring VA width may not accurately distinguish EVA from normal VA. Variations in the VA shape as shown in Fig. 4 are disregarded by classification systems based on VA diameter at fixed points, such as the Cincinnati criteria [19]. Therefore, such classification systems may easily lead to misclassified VA size, in particular if the distance of the VA is measured at only one point. 3D measurements consider the complete course of the VA as well as the length and height of the VA that are assumed to strongly impact the volume. Therefore, VA volume may be less prone to measurement errors and exhibits an excellent inter-rater variability. This is in line with the results of this study, where the 3D reconstruction showed better inter-rater reliability than VA width measurements indicating a more intuitive and reliable assessment. The median VA-volume in this study was 5.8 mm^3^ (95% CI 4.5–7.1 mm^3^) which is in accordance to another study investigating the VA-volume in controls [31]. Moreover, our results are in accordance with the diagnostic application of 3D measurement in other fields, e.g., the assessment of the growth of vestibular schwannomas, where more accurate values from volume measurements compared to two-dimensional measurements have been reported [32].

A volume threshold to distinguish EVA from normal VA may serve as an additional tool to 2D measurements in diagnosing EVA. The median volume of the VA_control_ in this study was only 5.2 mm^3^ which is comparable to values found in histological studies [31]. Based on the present study, we anticipate a value of approximately 15 mm^3^ to distinguish a normal VA from an EVA. With these data, future studies including clinical data may investigate possible associations between EVA as defined by 3D measurement and clinical symptoms, such as progressive hearing loss, vertigo or the estimated surgical risk, in particular the risk for a CSF gusher.

As a note of caution, however, EVA appears to be an etiologically distinct malformation with various factors that may account for the severity of the associated syndrome, i.e. EVAS [7, 33]. Although EVAS is an IEM, the malformation is not considered to be the cause of hearing loss. There is little evidence that either VA size correlates with the rate of hearing loss progression [34, 35] or that the frequency and severity of hearing loss is associated with the VA diameter determined at any point along the VA [36]. Moreover, most studies do not report VA size to be a predictor for the rate of hearing loss progression[37–42]. However, Berrettini et al. reported an association between a volume of the endolymphatic sac and endolymphatic sac complex greater than 1 mL and profound hearing loss [37]. It is assumed that the VA is enlarged secondary to an enlargement of the endolymphatic duct [8]. The enlargement of the bony VA, therefore, constitutes a “fossil-like record” of the primary cellular pathology of the endolymphatic compartment [8]. This is in line with largely missing correlations between the width of the VA and sudden/progressive hearing loss. Nevertheless, these studies were based on VA measurements evaluated on axial 2D images. Current studies provide evidence, that standardized measurements of the VA that are less prone to errors correlate with the probability of deafness [43]. For this reason, it may be worth revisiting these associations using VA volumetry to define EVA in both cases of isolated EVA as well as in cases of additional IEM. It has been shown in other studies, that standardized radiological measurements may give new insights into the etiopathology and prognosis of diseases associated with the endolymphatic duct and sac, such as Meniere’s disease [44–46]. When transferring such findings into a clinical context, algorithms for standardized detection of radiological features may gain importance [47]. The present study is limited by the sample sizes of the individual malformation types. Since the prevalence of the individual malformation types is low, statistical analyses are hindered by a small number of subjects. For this reason, cases of CH type I to type IV were summarized to allow a limited number of statistical tests. However, IEMs are rare and compared to other studies, the number of included data sets is high. Results regarding the clinical outcome such as gusher and the effect of cochlear implantation were missing, since the images were sent to the authors for second opinion. Another limitation is that MRI imaging was not assessed in this study. For this reason, only the bony covered VA but not the endolymphatic sac was reconstructed. Yet, this study was primarily designed to explore anatomic features in IEM with focus on the VA. Future studies may assess volume from MRI imaging to add a calculation of the volume of the intra-cranial portion of the endolymphatic sac and analyze larger patient groups including clinical data.

## Conclusions

Defining EVA based on VA diameter measurements has a high inter-rater variability. Furthermore, this method may fail to correctly diagnose EVA in cases with an abnormal shape of the VA. We introduce volumetry of the VA based on radiologic data as a novel tool to define EVA. Using this method of VA volumetry, we found a high prevalence of EVA in several types of IEM. VA volume correlates with the VA diameter, but may be less prone to misclassification of EVA as the VA diameter at defined points may be normal despite an abnormal shape and volume of the VA. 3D reconstruction allows an improvement in visualization and volumetric assessment of VA and may be an additional diagnostic tool in defining EVA.

## Abbreviations

CH: Cochlear hypoplasia
CSF: Cerebrospinal fluid
EVA: Enlarged vestibular aqueduct
HRCT: High-resolution CT
ICC: Intra-class correlation coefficient
IEM: Inner ear malformation
IPI: Incomplete partition type I
IPII: Incomplete partition type II
IRR: Inter-rater reliability
VA: Vestibular aqueduct
VA-V_control_: Vestibular aqueduct volume control
VA_operculum_: Vestibular aqueduct diameter at the operculum
VA_midpoint_: Vestibular aqueduct diameter at the midpoint
VA-V_CH_: Vestibular aqueduct volume cochlear hypoplasia
VA-V_EVAS_: Vestibular aqueduct volume enlarged vestibular aqueduct syndrome
VA-V_IPI_: Vestibular aqueduct volume incomplete partition type I
VA-V_IPII_: Vestibular aqueduct volume incomplete partition type II
